# Supplementary material for: In vivo pulse-chase in Caenorhabditis elegans reveals intestinal histone turnover changes upon starvation
Source: J Biol Chem. 2025 May 27;301(7):110299. doi: 10.1016/j.jbc.2025.110299 (PMC12221288; doi:10.1016/j.jbc.2025.110299)
Supplement: Supporting information [file mmc1.pdf]

Supplemental Figure Captions

Figure S1. Additional histone reporter data. (**A**) Representative images of anterior portion of histone reporter worm intestines stained with DAPI and Halo-Oregon Green (Halo-OG). Images shown as max intensity projection of confocal stack taken at 63X magnification. Scale bar, 25 µM. (**B**) Immunoblot comparing histone reporter expression between fed and starved strains. Synchronized, young adult histone reporter worms were either fed or starved for 6 hours prior to sample collection. 110 worms were collected for each sample (n=3). 3 dilutions of protein lysate were loaded for each sample. (**C**) Quantification of histone reporter protein expression between fed and starved strains. Quantification performed using ImageJ. Area of histone reporter band normalized to GAPDH loading control. Statistical significance was determined using a two-way ANOVA with Tukey's Multiple Comparison Test. ****p<0.0001; ns, not significant. (**D**) Amino acid sequences of the histone proteins used in this study. Amino acids highlighted in red of *his-72*/H3.3 reporter indicate sequence differences compared to *his-9*/H3.1.

Figure S2. In vivo labeling of an intestinal histone reporter with commercially available HaloTag ligands. (**A**) Representative images of *his-58*/H2B reporter worms pulse-labeled with either Halo-TMR, Halo-R110Direct, or Halo-Oregon Green. See **Results** and **Methods** for more details. Images shown as max intensity projection of confocal stack taken at 63X magnification under oil immersion. Scale bar, 25 µm. (**B**) Fluorescence quantitation of *his-58*/H2B worms pulse-labeled with Halo ligands. Significance was determined by performing a one-way ANOVA with Tukey's multiple comparisons test. *p<0.05; ***p<0.001; ****p<0.0001; ns, not significant.

Figure S3. Anterior nuclei are labeled most efficiently by soaking. (**A**) Representative images of *his-58*/H2B reporter worms pulse-labeled with 300 nM Halo-TMR and immediately fixed, stained with DAPI and Halo-Oregon Green, and imaged. Images shown as montage of max intensity projection of confocal stack taken at 40x magnification under oil immersion. Scale bar, 75 µm. (**B**) Quantitation of Halo-TMR fluorescence. Significance was determined by performing a one-way ANOVA with Tukey's multiple comparisons test. *p<0.05; ***p<0.001; ****p<0.0001; ns, not significant. (**C**) Summary statistics from one-way ANOVA with Tukey's multiple comparisons test on nuclei groupings from Halo-TMR pulse and No pulse control samples.

Figure S4. In vivo pulse-chase labeling of intestinal *his-9*/H3.1 histone reporter worms. (**A**) Representative images of fed and starved *his-9*/H3.1 reporter anterior intestinal nuclei after a 0- or 3-hour chase. Images shown as max intensity projection of confocal stack taken at 63X magnification under oil immersion. Scale bar, 10 µm. (**B-C**) Fluorescence intensity measurements of fed (B) and starved (C) *his-9*/H3.1 reporter anterior nuclei (see **Results** and **Methods**). Significance was determined by performing a one-way ANOVA with Tukey's multiple comparisons test. *p<0.05; ****p<0.0001; ns, not significant. (**D**) Representative images of fed and starved *his-58*/H2B and *his-9*/H3.1 no pulse control samples. Images shown as max intensity projection of confocal microscopy stack taken at 63X magnification. Scale bar, 10 µm.

Figure S5. LMN-1 reporter is more stable than H2B reporter using Halo tag *in vivo* pulse-chase method. (**A**) Linear diagram of the HaloTag LMN-1 reporter. An *elt-2* intestine-specific promoter drives expression of LMN-1 fused to a V5 epitope and HaloTag. The reporter has an *unc-54* 3'UTR and intergenic region. (**B**) HaloTag pulse-chase method. Reporter worms were pulse-labeled by soaking reporter worms in Halo-TMR ligand. Worms were either collected immediately (0 hr) or moved back to plates for 3 hours (3 hr). At each timepoint, worms were fixed and stained to visualize nuclei and total reporter protein. Worms were imaged on a confocal microscope and Halo-TMR fluorescence used as a measure of protein stability. (**C**) Representative fluorescent images of reporter worms collected at each timepoint stained with DAPI, Halo-Oregon Green (Halo-OG), and Halo-TMR. Scale bar, 10 µm. (**D**) Quantification and analysis of Halo-TMR fluorescence intensity in reporter worms. Significance was determined by performing a two-way ANOVA with Tukey's multiple comparisons test. *p<0.05; ****p<0.0001; ns, not significant.

Figure S6. Chromatin Immunoprecipitation analysis. Immunoblot of chromatin immunoprecipitation (ChIP) of histone reporter proteins. Input samples loaded as a 20% ratio of elute sample to evaluate efficiency of immunoprecipitation. Histone reporter proteins were successfully immunoprecipitated and enriched.

Table S1. ChIP-seq DNA metrics. (**A**) Quantification of DNA concentration in ChIP-seq experimental samples. DNA concentrations were comparable in all inputs. In IP samples, DNA was detected in histone samples but undetectable in N2 IP control samples. DNA quantification performed with a Qubit dsDNA high sensitivity kit. (**B**)

Raw read metrics from input samples filtered with Fastp and featureCounts programs. (**C**) Raw read metrics from IP samples filtered with Fastp and featureCounts programs.

Table S2. *his-9*/H3.1 reporter gene targets identified by ChIP-seq. 187 genes were identified as *his-9*/H3.1 targets in fed animals and 25 genes were identified as *his-9*/H3.1 targets in starved animals. All 25 starved targets were found in fed samples.

Table S3. Strains and plasmids used in this study.

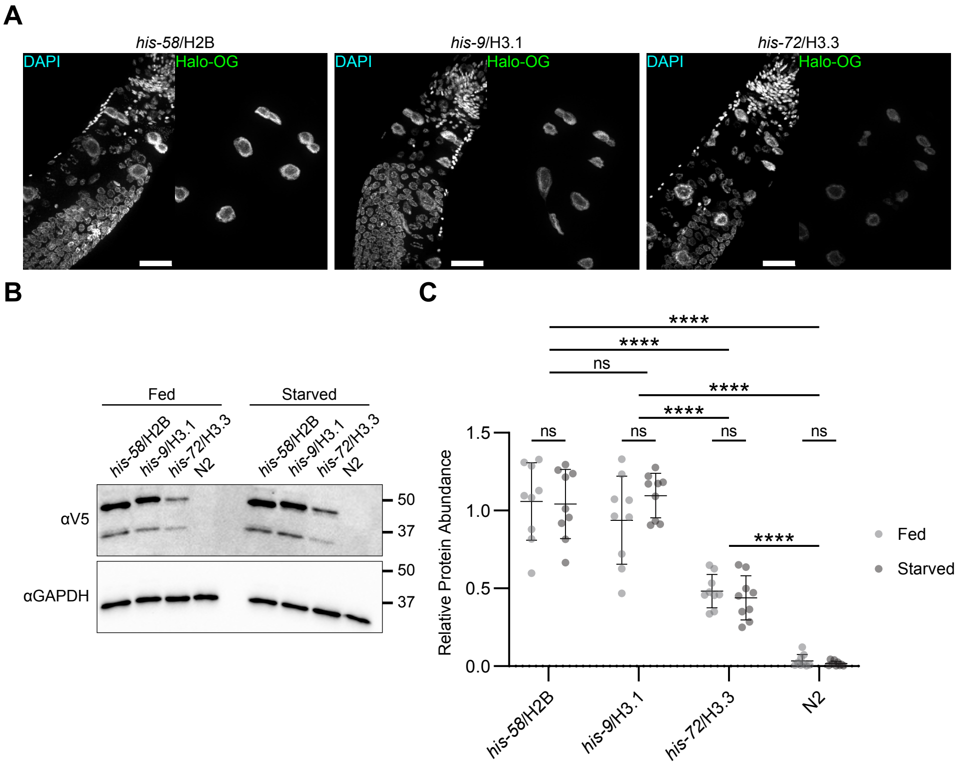

D

	10 · 20 · 30 · 40 · 50
his-58/H2B	MPPKPSAKGAKKAAKTVVAKPKDGKKRRHARKESYSVYIYRVLKQVHPDT
his-9/H3.1	MARTKQTARKSTGGKAPRKQLATKAARKSAPASGGVKKPHRYRPGTVALR
his-72/H3.3	MARTKQTARKSTGGKAPRKQLATKAARKSAPTTGGVKKPHRYRPGTVALR
	60 · 70 · 80 · 90 · 100
his-58/H2B	GVSSKAMSIMNSFVNDVFERIASEASRLAHYNKRSTISSREIQTAVRLIL
his-9/H3.1	EIRRYQKSTELLIRRAPFQRLVREIAQDFKTDLRFQSSAVMALQEAAEA
his-72/H3.3	EIRRYQKSTELLIRKLPFQRLVREIAQDFKTDLRFQSAAIGALQEASEA
	110 · 120 · 123 · 130 · 136
his-58/H2B	PGELAKHAVSEGTKAVTKYTSSK
his-9/H3.1	YLVGLFEDTNLCAIHAKRVTIMPKDIQLARRIRGERA
his-72/H3.3	YLVGLFEDTNLCAIHAKRVTIMPKDMQLARRIRGERA

Figure S2

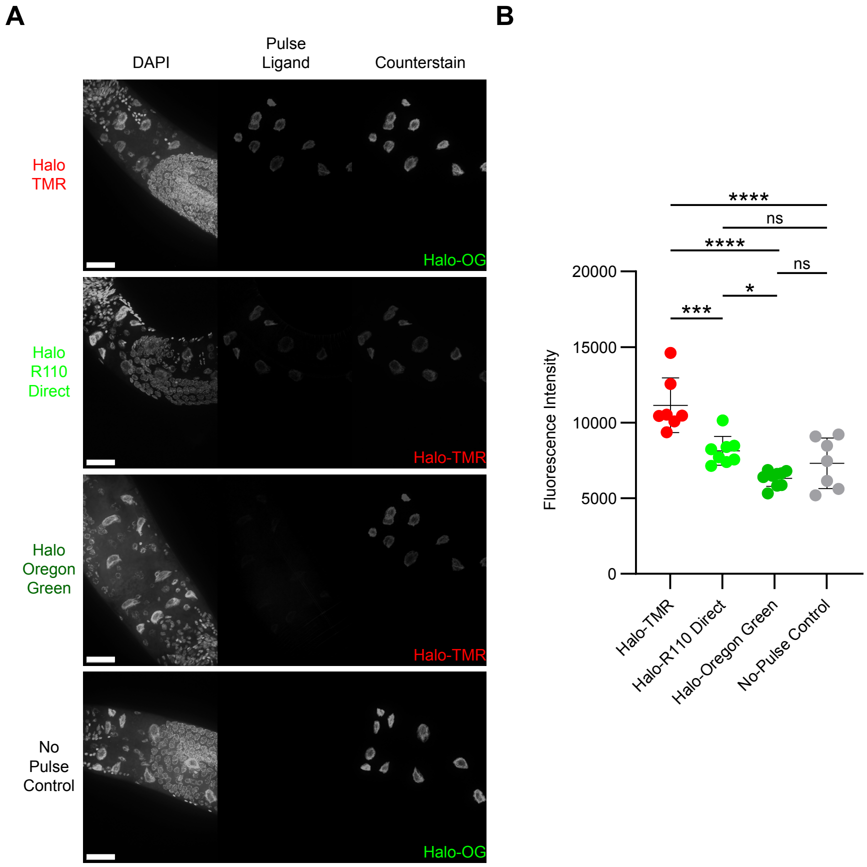

A
DAPI
Pulse Ligand
Counterstain
Halo TMR
Halo R110 Direct
Halo Oregon Green
No Pulse Control
Halo-OG
Halo-TMR
Halo-TMR
Halo-OG
B
Fluorescence Intensity
20000
15000
10000
5000
0
****
ns
****
ns
*
***
Halo-TMR
Halo-R110 Direct
Halo-Oregon Green
No-Pulse Control

A

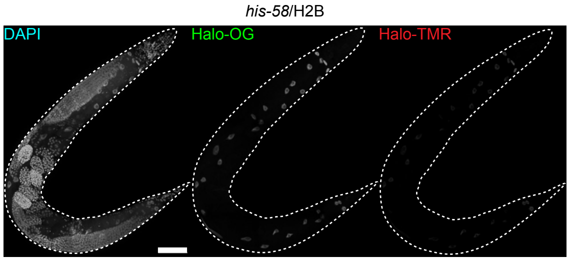

B

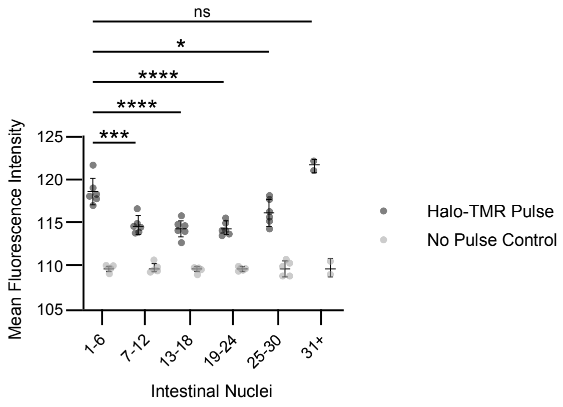

C

Halo-TMR Pulse Statistics

Nuclei Comparison	P Value
1-6 vs. 7-12	0.0001
1-6 vs. 13-19	<0.0001
1-6 vs. 19-24	<0.0001
1-6 vs. 25-30	0.0205
1-6 vs. 31+	0.0619
7-12 vs. 13-19	0.9934
7-12 vs. 19-24	0.9912
7-12 vs. 25-30	0.3149
7-12 vs. 31+	<0.0001
13-19 vs. 19-24	>0.9999
13-19 vs. 25-30	0.1194
13-19 vs. 31+	<0.0001
19-24 vs. 25-30	0.1112
19-24 vs. 31+	<0.0001
25-30 vs. 31+	0.0001

No Pulse Control Statistics

Nuclei Comparison	P Value
1-6 vs. 7-12	>0.9999
1-6 vs. 13-19	0.9975
1-6 vs. 19-24	>0.9999
1-6 vs. 25-30	>0.9999
1-6 vs. 31+	>0.9999
7-12 vs. 13-19	0.9911
7-12 vs. 19-24	0.9996
7-12 vs. 25-30	>0.9999
7-12 vs. 31+	>0.9999
13-19 vs. 19-24	0.9997
13-19 vs. 25-30	0.9952
13-19 vs. 31+	0.9968
19-24 vs. 25-30	>0.9999
19-24 vs. 31+	0.9998
25-30 vs. 31+	>0.9999

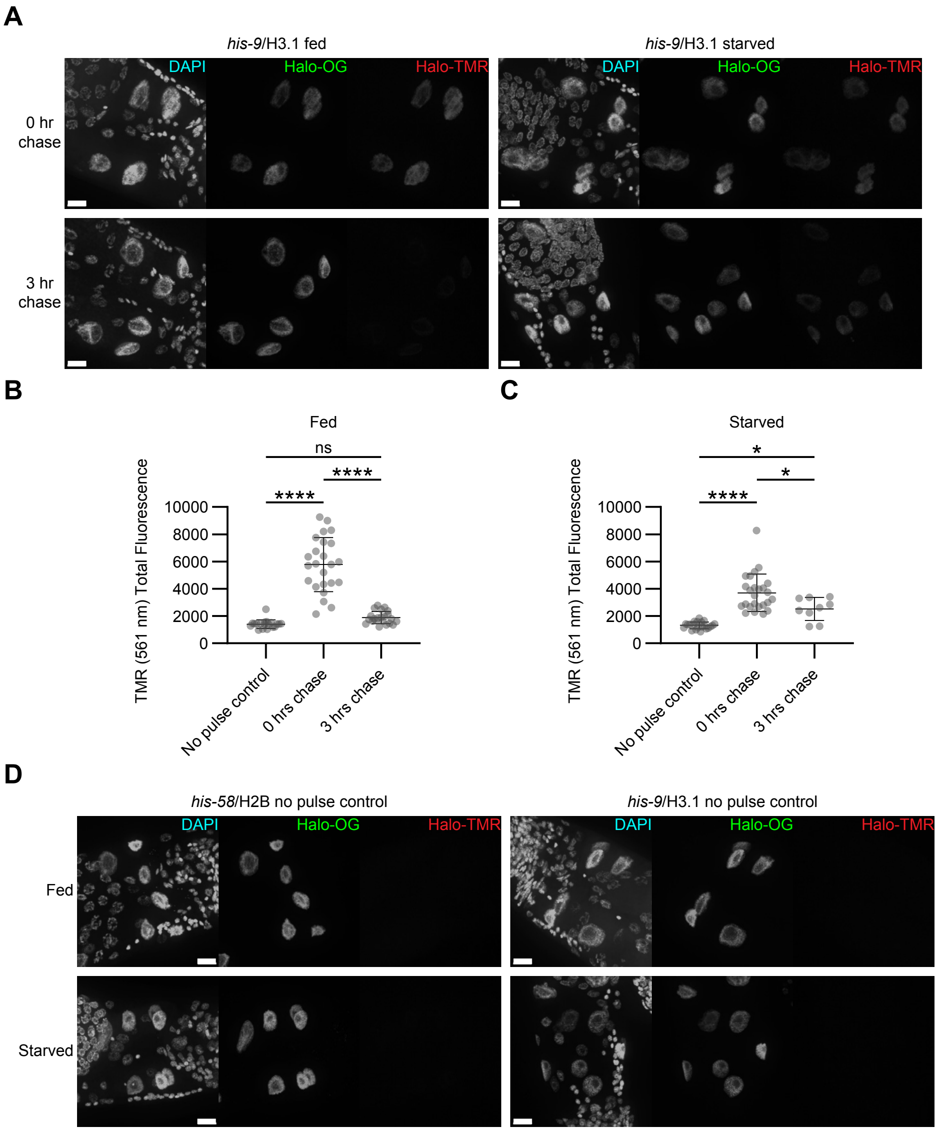
A
his-9/H3.1 fed
his-9/H3.1 starved
DAPI
Halo-OG
Halo-TMR
0 hr chase
3 hr chase
B
Fed
C
Starved
ns
****
*
TMR (561 nm) Total Fluorescence
10000
8000
6000
4000
2000
0
No pulse control
0 hrs chase
3 hrs chase
D
his-58/H2B no pulse control
his-9/H3.1 no pulse control
Fed
Starved

Figure S5

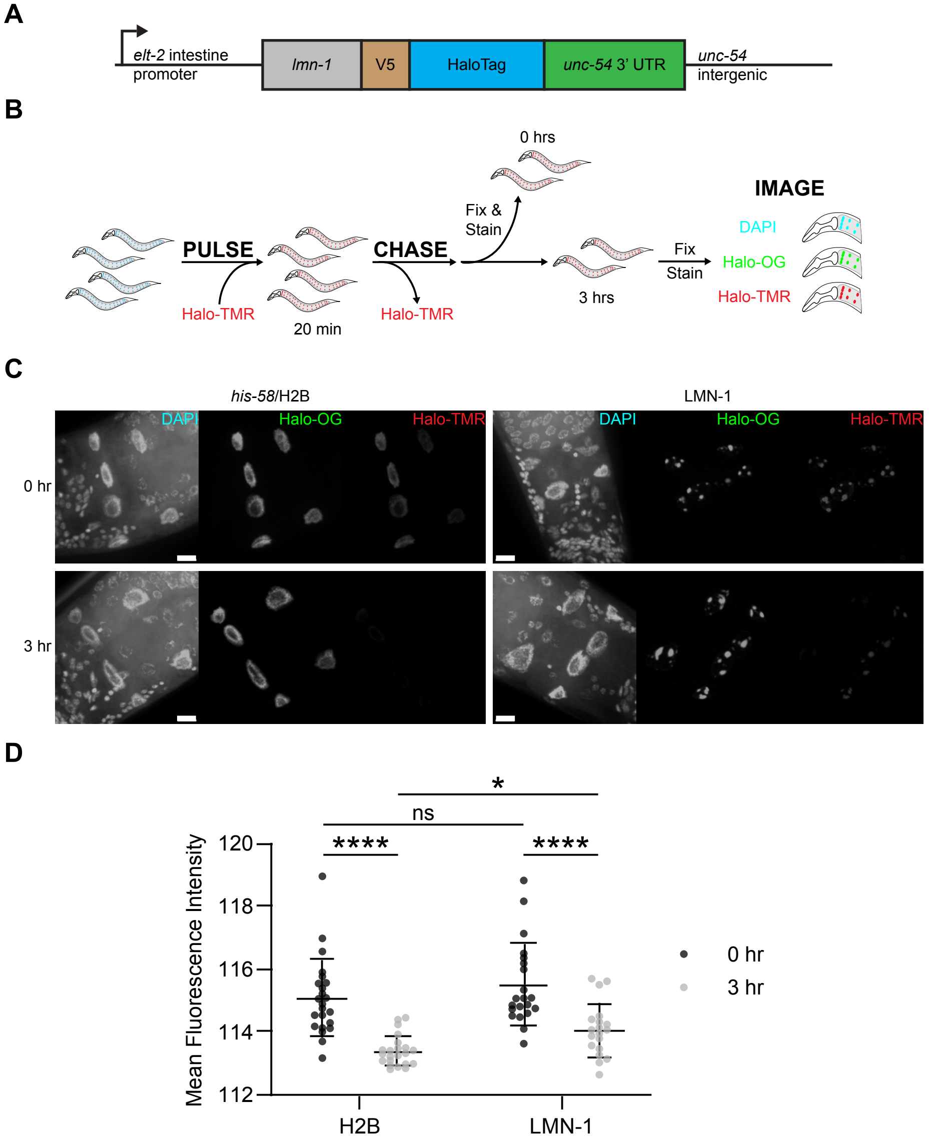

A
elt-2 intestine promoter
lmn-1
V5
HaloTag
unc-54 3' UTR
unc-54 intergenic
B
0 hrs
Fix & Stain
IMAGE
DAPI
Halo-OG
Halo-TMR
PULSE
CHASE
Halo-TMR
20 min
Halo-TMR
3 hrs
Fix
Stain
C
his-58/H2B
LMN-1
DAPI
Halo-OG
Halo-TMR
0 hr
3 hr
D
*
ns
****
****
Mean Fluorescence Intensity
120
118
116
114
112
H2B
LMN-1
0 hr
3 hr

Figure S6

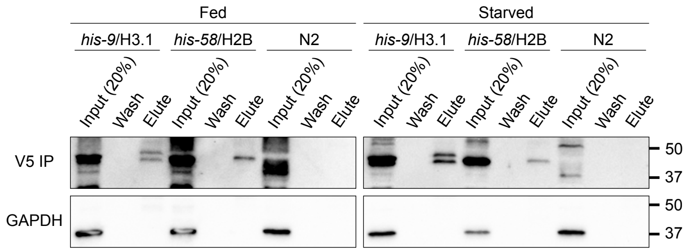

Fed
Starved
his-9/H3.1
his-58/H2B
N2
his-9/H3.1
his-58/H2B
N2
Input (20%)
Wash
Elute
V5 IP
GAPDH
50
37
50
37

Table S1

A

Sample	Input Concentration (ng/ul)	Elute Concentration (ng/ul)
H3.1_fed_1	39.3	0.101
H3.1_fed_2	16.2	0.057
H3.1_fed_3	30.2	0.065
H3.1_starved_1	37.2	0.1
H3.1_starved_2	26.4	Out of Range
H3.1_starved_3	31.2	0.15
H3.1_starved_4	7.9	0.093
H2B_fed_1	34.5	0.053
H2B_fed_2	20.9	0.07
H2B_fed_3	23.4	0.069
H2B_starved_1	38.9	0.054
H2B_starved_2	18.2	0.075
H2B_starved_3	17.7	0.076
H2B_starved_4	11.2	0.137
N2_IP_control_1	6.52	Out of Range
N2_IP_control_2	15.3	Out of Range
N2_IP_control_3	25.3	Out of Range

B

Sample	Reads before filtering (M)	Reads after filtering (M)	Duplication Rate	% Reads Passing Filters
his58_fed_rep_1	38.003455	37.595718	22.08%	98.93%
his58_fed_rep_2	20.880265	20.699801	15.72%	99.14%
his58_fed_rep_3	25.200334	24.967819	17.97%	99.08%
his58_starved_rep_1	46.433194	46.299141	21.21%	99.71%
his58_starved_rep_2	23.766361	23.669795	16.96%	99.59%
his58_starved_rep_3	21.686575	21.412442	17.33%	98.74%
his58_starved_rep_4	18.089458	17.984899	8.57%	99.42%
his9_fed_rep_1	61.853668	61.136978	25.86%	98.84%
his9_fed_rep_2	20.003081	19.761673	15.21%	98.79%
his9_fed_rep_3	0.389489	0.388096	11.12%	99.64%
his9_starved_rep_1	22.896645	22.825477	16.11%	99.69%
his9_starved_rep_2	9.350268	9.267513	12.47%	99.11%
his9_starved_rep_3	25.080913	24.871287	17.77%	99.16%
his9_starved_rep_4	14.777976	14.688478	7.40%	99.39%

C

Sample	Reads before filtering (M)	Reads after filtering (M)	Duplication Rate	% Reads Passing Filters
his58_fed_rep_1	95.872977	95.394471	30.69%	99.50%
his58_fed_rep_2	44.231096	44.001995	22.43%	99.48%
his58_fed_rep_3	42.391156	41.202747	21.75%	97.20%
his58_starved_rep_1	86.473105	85.877401	29.73%	99.31%
his58_starved_rep_2	40.902087	40.716067	23.87%	99.55%
his58_starved_rep_3	42.2955	41.647962	19.88%	98.47%
his58_starved_rep_4	27.746377	27.489943	15.84%	99.08%
his9_fed_rep_1	96.861723	96.362226	31.48%	99.48%
his9_fed_rep_2	49.92069	49.563775	23.63%	99.29%
his9_fed_rep_3	37.246509	36.813875	23.24%	98.84%
his9_starved_rep_1	63.173446	62.920141	28%	99.60%
his9_starved_rep_2	12.789964	12.667543	14.67%	99.04%
his9_starved_rep_3	43.54556	43.023264	20.91%	98.80%
his9_starved_rep_4	26.007827	25.859124	15.48%	99.43%
N2_IP_control_rep_1	8.678306	8.532422	17.57%	98.32%
N2_IP_control_rep_2	2.417762	2.287926	35.17%	94.63%

Table S2

***his-9*/H3.1 fed targets**		***his-9*/H3.1 starved targets**	**Overlapping targets**	***his-9*/H3.1 fed unique targets**	
smd-1	*K12H4.2*	*nep-17*	*nep-17*	*ech-7*	*ZK1248.17*
ech-7	*his-25*	*ccdc-149*	*ccdc-149*	*Y71A12C.2*	*C37C3.2*
Y71A12C.2	*F01D5.1*	*F54H5.3*	*F54H5.3*	*col-110*	*cytb-5.1*
nep-17	*C45B2.8*	*K04E7.1*	*K04E7.1*	*nhr-141*	*ctsa-3.1*
ccdc-149	*R12B2.7*	*smd-1*	*smd-1*	*K08D8.2*	*cyp-35C1*
ZC190.4	*str-7*	*dgat-2*	*dgat-2*	*klf-3*	*ZK1127.3*
C55B7.3	*daf-16*	*his-42*	*his-42*	*F01D5.2*	*srh-243*
his-72	*F22F7.11*	*W04D12.1*	*W04D12.1*	*C05D12.5*	*gpa-1*
col-110	*tatn-1*	*C46H11.7*	*C46H11.7*	*pdi-3*	*ZC190.5*
nhr-141	*pals-24*	*asp-1*	*asp-1*	*Y119D3B.21*	*gst-27*
dgat-2	*lec-9*	*ZC190.4*	*ZC190.4*	*clec-183*	*C47E8.11*
F54H5.3	*C44C11.6*	*C46H11.3*	*C46H11.3*	*M6.11*	*K10C2.12*
K08D8.2	*ZK6.11*	*pept-1*	*pept-1*	*F11E6.6*	*his-10*
klf-3	*adk-1*	*his-72*	*his-72*	*spp-21*	*his-53*
F01D5.2	*ZK1248.17*	*C55B7.3*	*C55B7.3*	*M163.1*	*hal-3*
F10C1.8	*C37C3.2*	*hpo-34*	*hpo-34*	*zip-12*	*K06A4.10*
C05D12.5	*cytb-5.1*	*act-5*	*act-5*	*C27D6.12*	*gstk-2*
pdi-3	*ctsa-3.1*	*his-44*	*his-44*	*F41H10.12*	*gdh-1*
his-42	*cyp-35C1*	*his-11*	*his-11*	*hpo-26*	*pqn-42*
Y119D3B.21	*ZK1127.3*	*F10C1.8*	*F10C1.8*	*F31E3.12*	*lap-2*
clec-183	*srh-243*	*ctsa-2*	*ctsa-2*	*ldp-1*	*fat-2*
pept-1	*gpa-1*	*pqm-1*	*pqm-1*	*eps-8*	*mul-1*
M6.11	*ZC190.5*	*his-15*	*his-15*	*ifb-2*	*acs-19*
F11E6.6	*gst-27*	*nspg-13*	*nspg-13*	*sre-44*	*pes-4*
spp-21	*C47E8.11*	*pals-23*	*pals-23*	*C17F4.12*	*srz-29*
his-44	*K10C2.12*			*nlp-36*	*nspg-7.1*
M163.1	*his-10*			*R10E8.1*	*C18B2.5*
pqm-1	*his-53*			*C05G5.7*	*F56C9.8*
his-15	*hal-3*			*sma-5*	*F48D6.4*
ctsa-2	*K06A4.10*			*spp-4*	*K06G5.1*
K04E7.1	*gstk-2*			*nlp-68*	*C29F7.3*
zip-12	*gdh-1*			*dmd-6*	*his-9*
C27D6.12	*pqn-42*			*ifc-1*	*F42A10.7*
F41H10.12	*lap-2*			*nhr-3*	*odc-1*
hpo-26	*fat-2*			*fil-2*	*F49F1.5*
F31E3.12	*mul-1*			*smut-1*	*hrpa-2*
ldp-1	*acs-19*			*C52B9.19*	*his-12*
C46H11.7	*pes-4*			*VZK822L.2*	*lec-1*
W04D12.1	*srz-29*			*elo-2*	*Y39G8B.9*
eps-8	*nspg-7.1*			*ins-37*	*F21F8.5*
ifb-2	*C18B2.5*			*C39B10.7*	*F09B12.3*
sre-44	*F56C9.8*			*fkh-9*	*cki-1*
C17F4.12	*F48D6.4*			*srh-269*	*Y32F6A.4*
nlp-36	*K06G5.1*			*C50B8.6*	*M02F4.2*
R10E8.1	*C29F7.3*			*bcat-1*	*str-206*
nspg-13	*his-9*			*eat-6*	*zip-3*
C05G5.7	*F42A10.7*			*nrfl-1*	*C34H4.1*
sma-5	*odc-1*			*crh-2*	*C05D12.3*
spp-4	*F49F1.5*			*tmem-189*	*ets-9*
nlp-68	*hrpa-2*			*nhr-31*	*T24B8.3*
pals-23	*his-12*			*his-26*	*vha-11*
dmd-6	*lec-1*			*frh-1*	*F15D3.5*
hpo-34	*Y39G8B.9*			*his-19*	*clec-50*
ifc-1	*F21F8.5*			*ifp-1*	*btb-12*
C46H11.3	*F09B12.3*			*K11C4.1*	*his-21*
nhr-3	*asp-1*			*spp-23*	*trx-3*
his-11	*cki-1*			*his-13*	*tald-1*
fil-2	*Y32F6A.4*			*csn-5*	*hsp-12.3*
smut-1	*M02F4.2*			*R12B2.6*	*clec-187*
C52B9.19	*str-206*			*R10E8.3*	*ZK1320.2*
VZK822L.2	*zip-3*			*kgb-2*	*ampd-1*
elo-2	*C34H4.1*			*nep-22*	*his-14*
ins-37	*C05D12.3*			*dod-19*	*T27E4.7*
C39B10.7	*ets-9*			*F01D5.3*	*Y46D2A.2*
fkh-9	*T24B8.3*			*pcp-3*	*rpl-3*
srh-269	*vha-11*			*B0348.10*	*F20G2.1*
C50B8.6	*F15D3.5*			*his-43*	*srx-41*
bcat-1	*clec-50*			*K12H4.2*	*F28A12.3*
eat-6	*btb-12*			*his-25*	*F28H7.2*
nrfl-1	*his-21*			*F01D5.1*	*vha-6*
crh-2	*trx-3*			*C45B2.8*	*bed-2*
tmem-189	*tald-1*			*R12B2.7*	*Y40C7B.4*
nhr-31	*hsp-12.3*			*str-7*	*nkb-3*
his-26	*clec-187*			*daf-16*	*T25C12.3*
frh-1	*ZK1320.2*			*F22F7.11*	*hlh-30*
his-19	*ampd-1*			*tatn-1*	*nhr-14*
ifp-1	*his-14*			*pals-24*	*F09B12.7*
K11C4.1	*T27E4.7*			*lec-9*	*cysl-3*
spp-23	*Y46D2A.2*			*C44C11.6*	*F09C8.1*
his-13	*rpl-3*			*ZK6.11*	*spp-17*
act-5	*F20G2.1*			*adk-1*	*C26B2.2*
csn-5	*srx-41*				
R12B2.6	*F28A12.3*				
R10E8.3	*F28H7.2*				
kgb-2	*vha-6*				
nep-22	*bed-2*				
dod-19	*Y40C7B.4*				
F01D5.3	*nkb-3*				
pcp-3	*T25C12.3*				
B0348.10	*hlh-30*				
his-43	*nhr-14*				
	F09B12.7				
	cysl-3				
	F09C8.1				
	spp-17				
	C26B2.2				

Table S3

A

Strain	Genotype	Description
N2 Bristol		Wildtype strain
EG6699	ttTi5605 II; *unc-119*(ed3) III; oxEx1578	MosSCI worm generation
CDE5	ddcSi1 [*elt-2* promoter::*his-9*::linker::1xV5::Halo opt with introns::*unc-54* 3'UTR and intergenic] II	*his-9* reporter strain
CDE6	ddcSi2 [*elt-2* promoter::*his-58*::histone linker::1xV5::Halo opt with introns::*unc-54* 3'UTR and intergenic] II	*his-58* reporter strain
CDE8	ddcSi4 [*elt-2* promoter::*his-72*::histone linker::1xV5::Halo opt with introns::*unc-54* 3'UTR and intergenic] II	*his-72* reporter strain
CDE142	ddcSi43 [*elt-2* promoter::*lmn-1*::histone linker::1xV5::Halo opt with introns::*unc-54* 3'UTR and intergenic] II	*lmn-1* reporter strain

B

Plasmid	Description
pCFJ151	Standard Multiple Cloning site vector to generate MosSCI inserts at ttTi5605 location
pGH8	Coinjection marker. mCherry expression in the nervous system
pCFJ90	Use as co-injection marker. Red mCherry fluorescence in the pharynx
pCFJ104	Red mCherry expression in the pharynx driven by the *myo-3* promoter
pCFJ601	Mos1 Transposase
pDDC14	MosSCI vector, *elt-2* promoter, *his-58* tagged with V5;Halo, *unc-54* 3'UTR and intergenic
pDDC15	MosSCI vector, *elt-2* promoter, *his-9* tagged with V5;Halo, *unc-54* 3'UTR and intergenic
pDDC16	MosSCI vector, *elt-2* promoter, *his-72* tagged with V5;Halo, *unc-54* 3'UTR and intergenic
pDDC171	MosSCI vector, *elt-2* promoter, *lmn-1* tagged with V5;Halo, *unc-54* 3'UTR and intergenic
